# The influence of adiposity on the interactions between strength, physical function and cognition among older adults in the Australian Diabetes, Obesity and Lifestyle (AusDiab) study

**DOI:** 10.1186/s12877-022-03033-3

**Published:** 2022-04-22

**Authors:** Niamh L. Mundell, Parneet Sethi, Kaarin J. Anstey, Helen Macpherson, David W. Dunstan, Steve F. Fraser, Robin M. Daly

**Affiliations:** 1 Institute for Physical Activity and Nutrition (IPAN), School of Exercise and Nutrition Sciences, Deakin University, Geelong, Australia; 2 Physical Activity Laboratory, Baker Heart and Diabetes Institute, Melbourne, Australia; 3 UNSW Ageing Futures Institute, University of New South Wales, Sydney, Australia; 4 Neuroscience Research Australia, Sydney, Australia

**Keywords:** Mobility, Cognitive function, Ageing, Overweight

## Abstract

**Background:**

Being overweight or obese may be associated with lower physical and cognitive function, but in late-adulthood (≥ 65 years) evidence is mixed. This study aimed to investigate how being overweight or obese affected interactions between muscle strength, function and cognition in Australians aged ≥ 50 years, and whether interactions varied according to age (i.e. ≥ 50–65 vs > 65 years).

**Methods:**

This study included 2368 adults [mean (standard deviation) age: 63 (7) years; 56% female] from the 2011/2012 Australian Diabetes, Obesity and Lifestyle (AusDiab) follow-up. Physical function was assessed via timed up-and-go (TUG) and muscle strength from knee extensor strength (KES). Cognition was assessed using Mini-Mental-State Exam (MMSE), Spot-the-Word (STW), California Verbal Learning Test (CVLT) and Symbol–Digit-Modalities Test (SDMT). Beta binomial regression was used to evaluate how being overweight or obese influenced strength, physical and cognitive function associations.

**Results:**

Being overweight or obese did not affect strength-cognition associations regardless of sex or age. With slower physical function; obese females showed better STW (odds ratio [OR] 95% CI]: 1.070 [1.016, 1.127], *P* = 0.011); obese men better MMSE (OR [95% CI]: 1.157 [1.012, 1.322], *P* = 0.033); and obese men aged > 65 better CVLT (OR [95% CI]: 1.122 [1.035, 1.217], *P* = 0.019) and MMSE (OR [95% CI]: 1.233 [1.049, 1.449], *P* = 0.017) compared to normal weight participants.

**Conclusion:**

Slower physical function was associated with better performance in some cognitive domains in obese, but not in non-obese adults aged ≥ 50 years. These findings suggest some benefits of obesity to aspects of cognition when physical function is slower, but longitudinal follow-up studies are needed.

**Supplementary Information:**

The online version contains supplementary material available at 10.1186/s12877-022-03033-3.

## Background

The physical, functional and cognitive declines associated with advancing age are known to share several neurodegenerative and cardiometabolic risk factors [1]. Obesity is also associated with physical and functional decline, and during mid-life (40–60 years) may increase dementia risk by up to 40% in later life (≥ 65 years) [2]. Adults of any age who are overweight or obese [body mass index (BMI) greater than 25.0 and 30 kg/m^2^, respectively] are advised to pursue weight loss to reduce the risk of common chronic diseases such as cardiovascular disease, type 2 diabetes (T2D), musculoskeletal and mental health conditions, and all-cause mortality [3]. For example, a meta-analysis of 30,206 adults with obesity showed weight loss interventions, compared to control, reduced all-cause mortality (risk ratio [95%CI]: 0.82 [0.71, 0.95]), which equated to six fewer deaths per 1000 adults [4]. However, there is some evidence for an “obesity paradox”, whereby overweight and obesity may offer a survival benefit and lower the risk of developing some conditions, such as dementia and Alzheimer’s Disease (AD) [5], in late-life (≥ 75 years). Conversely, a weight loss trajectory between mid-life and late-adulthood has been associated with a 14% and 20% greater risk of developing dementia and AD, respectively [5], and has been implicated in “cognitive frailty” whereby muscle loss induced through unintended weight-loss is coupled with cognitive and physical function decline [1]. In addition, normal age-related reductions in muscle and bone mass may conflate BMI as an obesity index in late-life [6]. These paradoxical findings suggest BMI alone does not reliably indicate health status in older adults.

It is well established that ageing is associated with a loss in muscle mass and strength and a deterioration in physical function (e.g. slow gait speed), all of which have been associated with impaired cognitive function [1] and an accelerated rate of decline in certain cognitive domains (e.g. processing speed) [7]. However, greater fat mass was associated with greater knee extension muscle strength in a cohort of 2,307 adults aged 70–79 years, which may in part stem from the additional muscle contractile work required during locomotion and activities of daily living in the presence of greater adiposity [8]. Whether preserved muscle strength and physical function is the underlying factor differentiating overweight or obese older adults who are cognitively intact from older adults with poorer cognitive capacity remains uncertain [2]. Age and sex may also influence these associations [2]. The seemingly paradoxical roles of being overweight or obese in regard to muscle strength, physical function and cognition may be better understood when the interaction of weight status is considered according to age and sex. Therefore, the primary aim of this study was to investigate whether being overweight or obesity influences the association between muscle strength and physical function with cognition in Australian males and females aged ≥ 50 years from the Australian Diabetes, Obesity and Lifestyle (AusDiab) study. A secondary aim was to evaluate whether any interactions between physical and cognitive function differed by age (≥ 50 -65 versus > 65 years).

## Methods

### Participants

The AusDiab study is an epidemiological prospective cohort study examining the prevalence, incidence and risk factors of cardiometabolic diseases and associated conditions with the baseline undertaken in 11,247 community dwelling adults aged ≥ 25 years at study entry (1999/2000), with follow-up data collection occurring in 2004–2005 (*n* = 6,537) and 2011/2012 (*n* = 4,614) [9]. At baseline, a stratified cluster sampling method was used including six Australian states and one territory, as defined by census collection districts [9]. Details of sample size determination and sample selection are described elsewhere [9]. For this study, data from the 2011/12 follow-up was used, as cognitive function, muscle strength and physical function were assessed at this time point only (*n* = 4,614), Participants’ aged ≥ 50 years were included in the analysis to coincide with the typical timing of the age-related losses in muscle strength and function [10]. This research was performed in accordance with the Declaration of Helsinki [11], and the study was approved by the International Diabetes Institute (now Baker Heart and Diabetes Institute) Ethics Committee. Written informed consent was obtained from all participants prior to participation.

Those who had missing data for these outcomes (knee extensor strength [KES]: *n* = 618, timed-up-and-go [TUG]: *n* = 10) and relevant covariates (highest level of education, *n* = 404; risk of CVD, combined measures of cardiovascular disease, blood lipid profile and blood pressure, *n* = 70; T2D status, *n* = 55; BMI, *n* = 1; total physical activity [PA], *n* = 51; Centre for Epidemiologic Studies Depression Scale [CESD], *n* = 45; smoking status, *n* = 53; health-related quality of life [HR-QoL], *n* = 23; alcohol intake, *n* = 243) were excluded from the analysis. Analyses were confined to complete-case data on key exposure and risk factor variables. There were 2,368 participants remaining in the subset (60% of possible total sample). As some participants did not complete all the cognitive tests, the number of observations for each cognitive test were as follows: Spot the Word [STW] (*n* = 2,299), California Verbal Learning Test [CVLT] (*n* = 2,337), Symbol Digit Modalities Test [SDMT] (*n* = 2,341) and Mini Mental State Examination [MMSE] (*n* = 967).

### Cognitive function

The MMSE (a dementia screening tool reflective of general cognitive abilities) was included in participants aged > 40 years, as reported previously [12], using clinically validated cut-off score below 24 on a 30-point scale determining cognitive impairment [13]. The STW test is a word based decision making task which was included as it correlates with verbal intelligence in adults (as measured by the Mill Hill Vocabulary score) [14]. Participants were required to correctly identify the word item when visually presented with 60-item pairs including one word and one non-word [14]. Scores ranged from 0 to 60, with a higher score indicative of better performance [14]. Immediate verbal memory was evaluated using the first trial of CVLT [15].The CVLT is reliable and validated test of verbal memory in adults [15]. Participants were read out a list of 16 nouns in a fixed order, and then asked to recall as many words as possible immediately. Scores ranged from 0 to 16 with a higher score indicative of better performance [15]. Processing speed was measured with the SDMT (oral version) [16]. Participants used a coded key to substitute nine abstract symbols to corresponding numerical digits for 90 s [16]. Scores ranged from 0 to 110, with higher scores indicative of better performance [16].

### Muscle strength and physical function

Muscle strength and physical function were assessed with the KES test and the 2.44 m TUG, respectively, as described in detail elsewhere [17]. Briefly, TUG is a validated and sensitive test of functional mobility in community dwelling older adults which incorporates aspects of gait speed, dynamic balance and lower limb strength [18]. TUG has demonstrated excellent validity and reliability in those with (*r* = 0.92, κ = 0.48) and without (*r* = 0.85, κ = 0.24) cognitive impairment [18]. For this test, participants were seated in a chair at the end of a 2.44 m walkway and the command ‘go’, were instructed to stand and walk at a comfortable speed around the cone, return to the chair and sit, with the time to complete the test recorded to the nearest millisecond (measured by stopwatch) [17]. Higher TUG scores (seconds) indicate worse performance (i.e., participants were slower to complete the task). Isometric KES was measured with the Lord’s strap assembly and strain gauge (Neuroscience Research Australia, Sydney, Australia). Participants were seated with 90-degrees of flexion at their hip and knee, and a strap fastened approximately 5–10 cm above the ankle of the dominant leg. After a practice trial and a 1 min rest, two test trials were conducted whereby participants were instructed to forcefully extend their leg against the strap for 2–3 s [19]. Results were reported in kg, with the highest score reported. The KES has shown good construct validity compared with other lower limb strength measures (*r* = 0.768) [19] and good test–retest reliability (ICC > 0.9) [20].

### Demographic, health and medical information

Information on age, sex, level of education and cardiovascular disease (CVD) history were collected by an interview administered questionnaire as previously reported [9]. Education was categorised as: (a) never, primary or some high school, (b) completed high school, year 12 or equivalent, or (c) completed university, technical and further education (TAFE) or equivalent. All participants undertook blood sampling, and those who were not being treated for T2D and females who were not pregnant undertook a standard 75 g oral glucose tolerance test [21]. Participants were classified as having T2D if they reported a previous medical diagnosis, were treated with hypoglycaemic medication, had fasting plasma glucose (FPG) ≥ 7 mmol/L or a 2-h plasma glucose (PG) ≥ 11.1 mmol/L [21]. Blood lipids were measured enzymatically (Olympus AU600 analyser) [9]. Hyperlipidaemia was classified as triglycerides ≥ 1.7 mmol/L or total cholesterol ≥ 5.5 mmol/L or LDL-cholesterol >  = 2.0 mmol/L or HDL-cholesterol ≤ 1.0 mmol/L [22]. Impaired glucose tolerance was classified as FPG 5.6–6.9 mmol/L and 2-h PG 7.8–11.0 mmol/L. Impaired fasting glucose was classified as FPG 6.1- 6.9 mmol/L and 2-h PG 7.8 mmol/L [9]. CVD risk was categorised by the presence of cardiovascular disease, hyperlipidaemia, hypertension, or when participants reported taking antihypertensive medication. Blood sample analyses were undertaken using methodology described elsewhere [9].

### Anthropometry

Height was measured with a stadiometer (without shoes) to the nearest 0.5 cm; weight was measured with digital scales (minimal clothing, without shoes) to the nearest 0.1 kg [9, 17]. BMI was calculated as weight (kg) divided by height (m^2^) [9]. Overweight was classified as a BMI of 25 to < 30 kg/m^2^ and obesity as a BMI ≥ 30 kg/m^2^ using the World Health Organization classifications and consistent with the Australian clinical practice guidelines [23].

### Physical activity

PA for the previous seven days were reported via interviewer administration of the Active Australia survey [24], a standardised observational instrument which has shown to be reliable (ICC = 0.59) and valid (criterion validity = 0.3) in adults [25]. Total PA was calculated by adding time spent walking to time spent in other moderate intensity activities (if continuous and > 10 min), and time spent in vigorous activities doubled (to account for increased energy expenditure) [24]. Those who completed a total of ≥ 150 min per week of moderate to vigorous PA were classified as ‘active’, and those completing < 150 min per week as ‘inactive’, in accordance with the Australian Physical Activity guidelines [26].

### Alcohol intake and smoking status

Alcohol intake (grams per day) was assessed using a validated food frequency questionnaire (FFQ; Cancer Council of Victoria, Version 2) [27]. Smoking status was classified as either: (a) current daily smoker, (b) ex-smoker (smoking less than daily for at least the last three months, but previously smoke daily), and (c) non-smokers (never smoked daily) [9].

### Blood pressure

Resting blood pressure (seated) was assessed using a standard mercury sphygmomanometer (Victoria) or Dinamap (other locations) [28]. The mean of the closest two of three readings taken at 1 min intervals was used [28]. Participants were classified as hypertensive if they reported use of anti-hypertensive medication and/or if systolic blood pressure was ≥ 140 mmHg or diastolic blood pressure was ≥ 90 mmHg [28].

### Depressive symptoms

Depressive symptoms were assessed using the CESD (0–20 points) and categorised as either no depressive symptoms (< 10 points), or mild to severe depressive symptoms (≥ 10 points) according to population based norms [29].

### Health-related Quality of Life (HR-QoL)

HR-QoL was assessed with the Short Form Health Survey 36 (SF-36) questionnaire (Version 1) [30]. The SF-36 questionnaire includes eight health-related sub-categories. The mean scores from each sub-category are used to calculate overall HR-QoL, with higher scores reflecting better quality of life. Two component scores were derived: (a) a physical component summary score ([PCS] derived from the domains of physical health, role limitations, pain and perceived general health), and (b) a mental component summary score (MCS) derived from the domains of vitality, social functioning, emotional role limitations and general mental health. All scores were reported according to guidelines for Australian norm-based scores, whereby each domain score has a mean of 50 points and a SD of 10 points [30]. The SF-36 has shown good reliability validity, and consistency measuring health perception in a general population (Cronbach's *a* > 0.85, *r* =  > 0.75) [31].

### Statistical analysis

Descriptive statistics for categorical variables were expressed as frequencies, while continuous variables were represented by the mean and SD when data followed a normal distribution or as the median and IQR when distribution deviated from normality. ANOVA, chi-square or Mann–Whitney U tests were used to test for any differences in continuous and categorical outcomes between males and females and participants excluded and included in the analysis. A score on a cognitive test means that the response equates to a correct response and 1 point is added to the total score. The cognitive performance scores (MMSE, STW, CVLT, SDMT) were bounded outcomes (total scores range between 0 and the maximum achievable total score for the specific test). The data were not normally distributed, violating a conditional assumption for linear regression [32]. We therefore used odds ratio to analyse our data [32]. Responses for each cognitive assessment were classified as a series of binary questions (correct or incorrect response) [32]. As scale items are interrelated related and therefore correlated, data were checked for overdispersion by constructing a likelihood-ratio test, which compared the likelihood of the beta binomial model to the likelihood of the binomial model [32]. A beta-binomial modelling approach was selected to estimate the odds (OR and 95% CI) of obtaining a higher score on each of the cognitive tests for one unit increase of each physical test when all other variables are held constant after adjusting for potential confounders [33]. The variables for muscle strength (KES) and physical function (TUG) tests were centred at their mean for better interpretability of the intercept. To assess the potential interaction effect of BMI classification on any muscle strength-cognition, or physical function-cognition associations, an interaction term between BMI status and KES/TUG was included in the beta binomial model. The interaction term (reported as OR 95% CI) indicates how much the effect of physical function and muscle strength to cognition differs between BMI categories in multiplicative terms, (i.e., interaction effect by which a 1-kg or 1 s increment in muscle strength and physical function respectively change the estimated odds of obtaining cognitive scores for the overweight and obese participants divided by the corresponding multiplicative factor for normal BMI participants). A positive effect (OR ≥ 1) indicates a higher likelihood of obtaining a score equivalent to one point in a cognitive test (holding all other covariates at their means) whereas an OR < 1.0 indicates a lower likelihood of obtaining a score in a given cognitive test, with each additional point obtained representing better cognition [34]. Thereafter, the multivariate model was adjusted for confounders that were known to be associated with cognitive function based on prior studies and theoretical considerations (age, sex, smoking status, PA levels, CVD risk, T2D status education, presence of depressive symptoms, alcohol intake and HR-QoL) [7, 35]. The multivariate analysis (Model A) was adjusted for age (years) and sex (male/female). Model B included covariates in Model A and additionally adjusted for smoking status (yes/no), total PA (active/inactive), CVD risk (yes/no) and T2D status (yes/no). Model C included covariates in Model A and B (with the exception of sex and age when data were stratified) and also adjusted for education, depressive symptoms (yes/no), alcohol intake (grams per day) and SF-36 mental and physical component scores (0–100 points). Participants were stratified by sex for each model based on established differences in cognition between males and females [35]. Stratification by age (50–65 vs > 65 years) was also considered for each model to address the secondary aims of this study.

Post-hoc analysis was conducted to determine the average probability of scoring on cognitive tests (with each additional point representing better cognitive performance) across the continuum of muscle strength and physical function tests for each of the BMI categories. To calculate the average predicted probability, a post estimation margins command was used. The probability was calculated for each case, using the case’s covariates (smoking status, PA levels, CVD risk, T2D status, education, presence of depressive symptoms, alcohol intake and HR-QoL), with TUG centred at the mean. Finally, though the survey used a complex sample design, we did not apply the survey commands to control for a cluster effect. All statistical analyses were performed using Stata SE 15.0 software (Stata Corp, College Station, TX, USA). Statistical significance was set at *P* < 0.050 (two-tailed).

## Results

The characteristics of the 2,368 participants included in the study are shown in Table 1. Mean (SD) BMI was 27.3 (6.0) kg/m^2^, (range 14.3–58.0) (kg/m^2^) with 1,019 (43%) and 659 (28%) participants classified as overweight or obese, respectively. Less than 1% of participants were classified as underweight (BMI < 18.5 kg/m2 *n* = 11 [0.47%]) and these were grouped with normal BMI participants. Overall, included participants were more likely to be classified as physically active (64% vs 54%, *P* < 0.001), have fewer depressive symptoms (10% vs 12%, *P* < 0.001), completed high school or tertiary education (44% vs 40%, *P* < 0.001) and were more likely to be overweight or obese (71% vs 66%, *P* < 0.001) than those excluded. Additionally, those included were less likely to be current smokers (5% vs 8%, *P* < 0.001) or have T2D (10% vs 14%, *P* < 0.001). On average, increasing levels of adiposity were associated with a lower MMSE scores for females and lower STW for males (Supplementary Table 1).

**Table 1:** Characteristics of the total cohort (*n* = 2,368) and males (*n* = 1,050) and females (*n* = 1,318)

Variable	Total	Males	Females	*P*-value
Age, years^a^	62.6 (12.3)	63.2 (12.5)	62.1 (12.4)	0.0171
50–65	1,425 (60%)	619 (59%)	806 (61%)	0.277
> 65	943 (40%)	431 (41%)	512 (39%)	
Weight, kg^a^	77.5 (20.9)	84.5 (18.1)	71.3 (18.6)	** < 0.001**
Height, cm^a^	167.5 (14.0)	175.9 (9.5)	162.5 (8.6)	** < 0.001**
Body mass index, kg/m^2a^	27.3 (6.0)	27.5 (5.2)	27.1 (6.9)	**0.0424**
Normal, n (%)	690 (29%)	265 (25%)	425 (33%)	** < 0.001**
Overweight, n (%)	1,019 (43%)	510 (49%)	509 (39%)	
Obese, n (%)	659 (28%)	275 (26%)	384 (29%)	
SF-36 MCS, (1–100 points) ^a^	62.2 (15.7)	62.5 (14.7)	61.9 (16.3)	0.2114
SF-36 PCS, (1–100 points) ^a^	50.5 (11.0)	50.8 (9.8)	50.2 (11.9)	**0.0631**
Highest level of education				
None/some high school	1325 (56%)	568 (54%)	757 (57%)	0.104
High school/Tertiary	1043 (44%)	482 (46%)	561 (43%)	
Alcohol, g/d^a^	7.3 (20.0)	13.7 (26.7)	3.5 (12.7)	** < 0.001**
Smoking status, n (%)				
Current smoker	129 (5%)	63 (6%)	66 (5%)	** < 0.001**
Ex-smoker	858 (36%)	467 (45%)	391 (30%)	
Non-smoker	1381 (58%)	520 (50%)	861 (65%)	
Diabetes status, n (%)				
Normal	1757 (74%)	712 (68%)	1045 (79%)	** < 0.001**
Diabetes	242 (10%)	135 (13%)	107 (8%)	
Impaired fasting glucose	369 (16%)	203 (19%)	166 (13%)	
Cardiovascular disease risk, n (%)				
Yes	1181 (50%)	593 (56%)	588 (45%)	** < 0.001**
No	1187 (50%)	457 (44%)	730 (55%)	
Physical activity, min/wk ^a^	240 (420)	270 (480)	210 (360)	** < 0.001**
Sufficient, n (%)	1515 (64%)	699 (67%)	816 (62%)	0.050
Insufficient, n (%)	609 (26%)	246 (23%)	363 (28%)	
Depressive symptoms, n (%)				
No	2144 (90%)	962 (92%)	1182 (90%)	0.119
Mild	133 (6%)	57 (5%)	76 (6%)	
Severe	91 (4%)	31 (3%)	60 (4%)	
CESD score	0.10 (0.29)	0.08 (0.28)	0.10 (0.30)	0.1095
Income per year, n (%)				
≥ $80,000,	838 (39%)	446 (44%)	392 (34%)	** < 0.001**
$40,000–79,999	636 (29%)	285 (28%)	351 (30%)	
$30,000–39,999	229 (11%)	106 (11%)	123 (11%)	
$20,000–29,999	240 (11%)	96 (10%)	144 (12%)	
$10,000–19,999	185 (9%)	60 (6%)	125 (11%)	
$0–9,999	31 (1%)	8 (1%)	23 (2%)	
Lower leg muscle strength, kg^a^	23.3 (17.0)	30.4 (19.4)	18.9 (13.6)	** < 0.001**
Timed-up-and-go, sec^a^	5.84 (1.72)	5.66 (1.72)	6.02 (1.76)	** < 0.001**
Cognitive Reserve [STW (0–60)] ^a^	51 (7)	50 (7)	51 (6)	0.0628
Processing Speed [SDMT (0–110)] ^a^	50 (13)	48 (13)	51 (12)	** < 0.001**
Verbal Learning [CVLT (0–16)] ^a^	6 (3)	6 (3)	7 (4)	** < 0.001**
Global Cognition [MMSE (0–30)] ^a^	29 (1)	28 (2)	29 (2)	** < 0.001**

Data are number (%) or mean (standard deviation) when normally distributed. ^a^Median (interquartile range) when not normally distributed. *CVLT* California Verbal Learning Test (*n* = 2,337), *MMSE* Mini Mental State Exam (*n* = 967), *SDMT* Symbol–Digit Modalities Test (*n* = 2,341), *STW* Spot-the-Word (*n* = 2,299) Significant between group difference ***P* < 0.05**

### Muscle strength, physical function and cognition associations stratified by sex and age

No significant associations were seen between muscle strength and any measure of cognition for the total sample after adjustment for potential confounders, with the exception of MMSE which was inversely associated with muscle strength for females (OR [95%CI] 0.988 [0.977, 0.999]). Age stratification showed that this association remained significant only for females aged 50–65 years (OR [95%CI] 0.982 [0.966, 0.988]). Notably, for our muscle strength measurement, the maximum detectable strength was 60 kg. While 2% of the sample reached 60 kg, when these data were removed from analyses results were unchanged.

In females collectively, significant positive associations were shown between physical function and MMSE (OR [95%CI] 0.945 [0.895, 0.998]), STW (OR [95%CI] 0.971 [0.950, 0.993]) and SDMT (OR [95%CI] 0.969 [0.957, 0.982]). Age stratification showed these associations remained significant for CVLT in females aged 50–65 (OR [95%CI] 0.956 ([0.922, 0.991]).

Significant positive associations were observed in males collectively between physical function (lower scores equate to better performance) and MMSE (OR [95%CI] 0.912 [0.858, 0.968]); CVLT (higher scores equate to better performance) (OR [95%CI] 0.956 [0.932, 0.980]); and SDMT (OR [95%CI] 0.962 [0.949, 0.974]). When stratified by age, these associations remained significant for CVLT in males aged ≥ 65 yr (OR [95%CI]0.942 [0.907 0.978]).

### Muscle strength, physical function and cognition interactions with BMI status stratified by sex

Overall, there were no differences in the interactions between muscle strength (KES) and any measure of cognition by BMI categories for men or women in age adjusted analyses or with age stratification (50–65 vs > 65 years) (Table 2). The factor by which the odds of scoring 1 point in a cognitive test change for each of the BMI categories when muscle strength or physical function outcomes change by 1 unit are shown in Supplementary Tables 2 & 3 respectively.

**Table 2:** Ratio of the multiplicative factor (95% CI) i.e. interaction effect by which the estimated odds change given a 1-kg increase in muscle strength for the overweight and obese participants divided by the corresponding multiplicative factor for normal BMI participants for different sex and age categories

	**Adjusted odds ratio at the means for the BMI categories**					
	**Male**	**Female**	**Male, 50-65 yr**	**Male, > 65 yr**	**Female, 50-65 yr**	**Female, > 65 yr**
**Verbal Memory (California Verbal Learning Test)**						
Overweight	1.003 (0.993, 1.006)	0.999 (0.991, 1.007)	0.999 (0.991, 1.007)	0.999 (0.985, 1.013)	0.998 (0.989, 1.008)	0.995 (0.981, 1.009)
Obese	1.003 (0.995, 1.010)	1.000 (0.992, 1.009)	0.997 (0.988, 1.006)	1.008 (0.994, 1.023)	1.004 (0.993, 1.014)	0.989 (0.973, 1.006)
**Cognitive Reserve (Spot-the-Word)**						
Overweight	1.000 (0.992, 1.007)	0.998 (0.989, 1.007)	0.999 (0.991, 1.008)	0.998 (0.982, 1.014)	0.995 (0.985, 1.005)	1.006 (0.988, 1.024)
Obese	1.003 (0.995, 1.011)	0.999 (0.989, 1.008)	1.003 (0.994, 1.013)	1.003 (0.987, 1.020)	1.000 (0.989, 1.011)	1.000 (0.980, 1.021)
**Processing Speed (Symbol–Digit Modalities Test)**						
Overweight	1.000 (0.997, 1.004)	1.001 (0.997, 1.006)	0.999 (0.995, 1.004)	1.006 (0.999, 1.013)	1.000 (0.995, 1.005)	1.000 (0.990, 1.01)
Obese	1.000 (0.996, 1.004)	1.001 (0.996, 1.006)	0.998 (0.993, 1.003)	1.005 (0.998, 1.013)	1.002 (0.996, 1.008)	1.001 (0.990, 1.011)
**Global Cognition (Mini Mental State Exam)**						
Overweight	1.006 (0.987, 1.024)	1.012 (0.986, 1.039)	1.006 (0.974, 1.040)	1.008 (0.984, 1.032)	1.012 (0.972, 1.053)	1.007 (0.974, 1.041)
Obese	0.992 (0.973, 1.012)	0.996 (0.971, 1.022)	0.985 (0.950, 1.022)	0.997 (0.973, 1.022)	1.009 (0.971, 1.049)	0.988 (0.954, 1.023)

The results presented are OR (95%CI) for Model C adjusted for sex, age, smoking status, total physical activity, cardiovascular disease risk, type 2 diabetes status, education, depression, alcohol intake, SF-36 mental and physical component scores. The reference category is participants with normal BMI. ***P* < 0.050.** The sample size for each stratification is given below

**Verbal memory test -**Total sample: 2337; Male: 1037; Female: 1300; Male 50–65 yrs: 615; Male > 65 yrs: 422; Female 50–65 yrs: 800; Female > 65 yrs: 500

**Spot-the-Word -**Total sample: 2299; Male: 1017; Female: 1282; Male 50–65 yrs: 607; Male > 65 yrs: 410; Female 50–65 yrs: 792; Female > 65 yrs: 490

**Symbol–Digit Modalities Test -**Total sample: 2341; Male: 1035; Female: 1306; Male 50–65 yrs: 614; Male > 65 yrs: 421; Female 50–65 yrs: 802; Female > 65 yrs: 504

**Mini Mental State Exam -**Total sample: 967; Male: 442; Female: 525; Male 50–65 yrs: 154; Male > 65 yrs: 288; Female 50–65 yrs: 171; Female > 65 yrs: 354

**Fig. 1:**
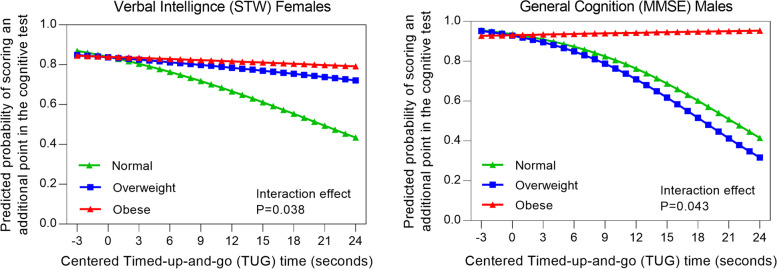
Predicted odds of obtaining an additional point (better performance) on verbal intelligence in females (**a**) and general cognition in males (**b**) for each second increase (poorer performance) in TUG time (centred at 6 s) by BMI categories (normal weight, overweight and obesity)

Sex stratified analyses indicated that a) for females overall, the odds of scoring (or showing better performance) on STW was higher in obese compared to normal weight women as TUG time increased (poorer performance) (Table 3). For instance, when TUG time increased by 1 s (indicating a decrease in performance), the change in the odds of scoring an additional point (or showing better performance) in STW was higher for obese versus normal weight females [OR = 1.070 (95% CI: 1.016, 1.127)] in model C] (Table 3); b) for males overall, odds of showing better MMSE was higher in obese compared to normal weight men as TUG time increased (poorer performance) (Table 3). For every 1-s increase in TUG time (indicating poorer performance), the change in the odds of scoring (or showing better performance) in MMSE [OR = 1.157, (95% CI: 1.012, 1.322)] was higher in obese compared to normal weight men, with a trend towards significance in CVLT (OR [95% CI]: 1.056 [1.000, 1.115], *P* = 0.051). Post-hoc analysis showed with poorer performance in TUG (longer duration), the average predicted probability of scoring was significantly lower for those with normal BMI compared to overweight and obese participants in STW (females) and MMSE (males) (Fig. 1). This indicates that an obese BMI classification interacts positively with associations between physical function and cognition for women (STW), and men (MMSE) > 50 years compared to overweight or normal BMI classification.

**Table 3:** Ratio of the multiplicative factor (95% CI) by which the estimated odds change given a 1-s increase in physical function test for the overweight and obese participants divided by the corresponding multiplicative factor for normal BMI participants for different sex and age categories

	**Adjusted odds ratio for scoring on the cognition test**					
	**Male**	**Female**	**Male, 50-65 yr**	**Male, > 65 yr**	**Female, 50-65 yr**	**Female, > 65 yr**
**Verbal Memory (CVLT)**						
Overweight	1.051 (0.994, 1.112)	1.031 (0.976, 1.089)	1.002 (0.924, 1.085)	1.063 (0.972, 1.163)	1.064 (0.975, 1.161)	1.012 (0.934, 1.096)
Obese	**1.056 (1.000, 1.115)**	1.029 (0.980, 1.081)	1.004 (0.921, 1.096)	**1.122 (1.035, 1.217)**	1.022 (0.940, 1.111)	1.023 (0.956, 1.094)
**Cognitive Reserve (Spot-the-Word)**						
Overweight	1.032 (0.974, 1.094)	1.054 (0.993, 1.118)	1.017 (0.933, 1.109)	1.063 (0.972, 1.161)	1.069 (0.976, 1.172)	1.052 (0.959, 1.155)
Obese	1.026 (0.975, 1.081)	**1.070 (1.016, 1.127)**	1.020 (0.930, 1.118)	1.031 (0.962, 1.106)	1.089 (0.998, 1.189)	1.062 (0.985, 1.145)
**Processing Speed (Symbol–Digit Modalities Test)**						
Overweight	1.001 (0.972, 1.030)	0.989 (0.958, 1.021)	0.998 (0.953, 1.045)	0.966 (0.927, 1.007)	0.985 (0.938, 1.034)	1.000 (0.948, 1.054)
Obese	1.011 (0.983, 1.040)	0.998 (0.969, 1.027)	1.026 (0.976, 1.078)	1.004 (0.968, 1.042)	0.980 (0.935, 1.027)	1.006 (0.961, 1.053)
**Global Cognition (Mini Mental State Exam)**						
Overweight	0.987 (0.879, 1.109)	1.008 (0.874, 1.163)	0.840 (0.630, 1.121)	0.992 (0.873, 1.129)	1.024 (0.731, 1.434)	0.986 (0.832, 1.169)
Obese	**1.157 (1.012, 1.322)**	1.009 (0.894, 1.137)	0.960 (0.732, 1.258)	**1.233 (1.049, 1.449)**	1.090 (0.803, 1.479)	0.988 (0.860, 1.136)

The results presented are OR (95%CI) for Model C adjusted for sex, age, smoking status, total physical activity, cardiovascular disease risk, type 2 diabetes status, education, depression, alcohol intake, SF-36 mental and physical component scores. The reference category is participants with normal BMI.* ***P* < 0.050.** The sample size for each stratification is given below

**Verbal memory test -**Total sample: 2337; Male: 1037; Female: 1300; Male 50–65 yrs: 615; Male > 65 yrs: 422; Female 50–65 yrs: 800; Female > 65 yrs: 500

**Spot-the-Word -**Total sample: 2299; Male: 1017; Female: 1282; Male 50–65 yrs: 607; Male > 65 yrs: 410; Female 50–65 yrs: 792; Female > 65 yrs: 490

**Symbol–Digit Modalities Test -**Total sample: 2341; Male: 1035; Female: 1306; Male 50–65 yrs: 614; Male > 65 yrs: 421; Female 50–65 yrs: 802; Female > 65 yrs: 504

**Mini Mental State Exam -**Total sample: 967; Male: 442; Female: 525; Male 50–65 yrs: 154; Male > 65 yrs: 288; Female 50–65 yrs: 171; Female > 65 yrs: 354

### Muscle strength, physical function and cognition interactions with BMI status stratified by sex and age

**Fig. 2:**
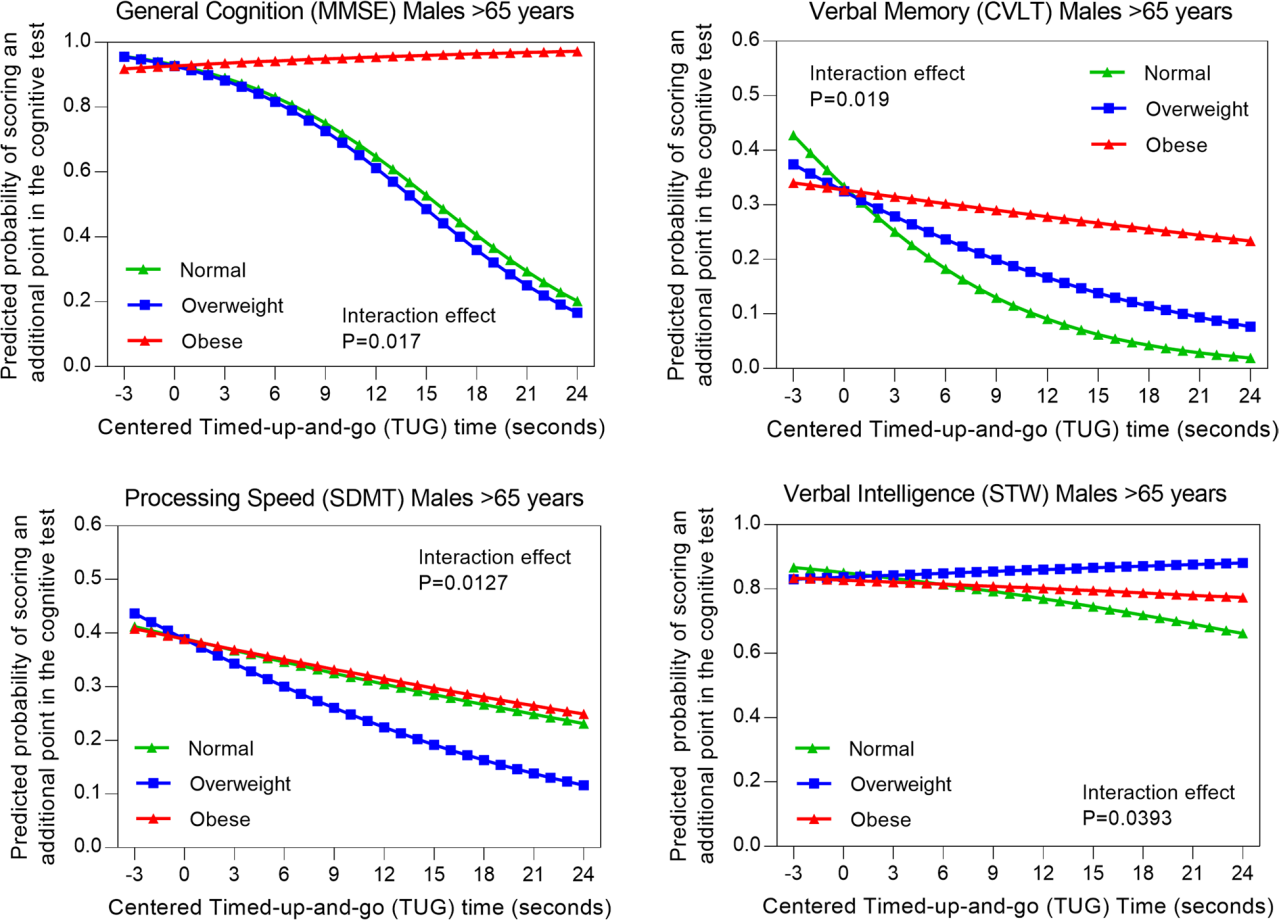
Predicted odds of obtaining an additional point (better performance) on general cognition (**a**), verbal memory (**b**), processing speed (**c**), and verbal intelligence (**d**) for each second increase (poorer performance) in TUG (centred at 6 s) by BMI categories (normal weight, overweight and obese) in males aged > 65 years

There were no differences in the interactions between muscle strength with any measure of cognition by BMI categories for either men or women when stratified by age (50–65 vs > 65 years). With regards to interactions between physical function and cognition, sex and age stratification showed: a) in males aged > 65 years, the odds of scoring (or showing better performance) on MMSE [OR = 1.233 (95% CI: 1.049, 1.449)] and CVLT [OR = 1.122 (95% CI: 1.035, 1.217)] was higher in obese compared to normal weight men as TUG time increased (poorer performance) (Table 3). Post-hoc analysis for average predicted probability showed the lower probability of scoring in CVLT and MMSE with poorer performance in TUG was more pronounced in normal BMI participants for males > 65 years of age compared to the overweight and obese categories (Fig. 2) These outcomes indicate that an obese BMI classification interacts positively with associations between physical function and cognition (MMSE, CVLT) in men aged > 65 years compared to normal BMI classification.

## Discussion

In this cohort of Australian middle-aged/older adults, a sex and age-group specific interactive effect of obesity was noted for the associations between physical function, but not strength, and some cognitive domains. When sex and BMI classification were considered: (i) there were no associations between lower limb muscle strength and cognitive function in males or females after adjustment for confounding variables (age, sex, smoking status, PA levels, CVD risk, T2D status education, presence of depressive symptoms, alcohol intake and HR-QoL); (ii) obese females > 50 years old showed significantly higher probabilities of scoring (better performance) on STW compared to normal and overweight females when physical function was slower; and (iii) obese males > 50 years old showed significantly higher probabilities of scoring in MMSE (better performance) when physical function was slower in adjusted analyses. When sex, BMI classification and age stratification were considered, there were no differences in the probabilities of better performance in any of the cognitive outcomes with poorer performance in physical function for males or females, except for MMSE and CVLT for obese men aged > 65 years. All findings remained significant after adjustment for several factors known to influence cognitive function, including smoking status, physical activity levels, cardiometabolic and psychosocial factors.

A key finding from our study was that the association between strength and cognition was not significant after adjusting for BMI, regardless of sex or age. Similarly, a study including 306 community-dwelling adults (mean age:73.6) showed no associations between lower limb strength (KES) and cognitive impairment classification determined with MMSE scores, however this study did not adjust for BMI, or include sex and age stratification [36]. In our study, prior to adjustment for BMI classification there were no strength-cognition associations shown in males, and an inverse association with MMSE in females aged 50 – 65 years. This contrasts with research showing stronger associations between upper limb strength and cognitive impairment (MMSE) in males compared to females, and in adults aged ≥ 70 years compared to aged 60–70 years (*n* = 1009) when adjusted for social, educational lifestyle (physical exercise, drinking, smoking), BMI, and self-reported chronic diseases [37]. However, strength-cognition associations may vary by muscle group, for example grip strength only explains approximately 40% of the variance in lower extremity strength [38], which reduces comparability with our results.

Prior research has shown positive associations between lower limb strength and cognitive performance (reaction-time) in women aged ≥ 60 (*n* = 202, mean age 72 years) [39]. Our contrasting findings may in part be explained by differing methodologies and measures of cognition, however it is possible that the compromising effect of greater adiposity outweigh any potential cognitive advantages of greater strength in overweight and obese older adults [40]. Additionally, more cognitively complex measures of physical function may reflect engagement in lifestyle behaviours promoting mobility and independence and therefore be more closely related to cognition in older adults than muscle strength [41, 42]. Furthermore, the MMSE may have greater sensitivity in late-adulthood and thereby correlate with the relatively more complex physical function test compared to during mid-life. However, post-hoc exploratory sensitivity analysis conducted in male participants revealed that when participants classified as cognitively impaired (MMSE < 25) and aged > 65 years were excluded (*n* = 19), results remained unchanged.

Our results showed normal weight and overweight female participants obtained significantly lower scores for STW (reflective of poorer verbal intelligence) with slower physical function compared to obese females aged ≥ 50 years. These results suggest that for females aged ≥ 50 years, obesity was associated with more stable STW scores even if physical function was slower. In contrast, normal and overweight females appeared more vulnerable than obese females to slower physical function regardless of STW scores. [6] STW measures word knowledge or verbal intelligence which has been associated with socioeconomic status, vulnerability to health problems and participation in healthy lifestyle behaviours [43]. STW is proposed to be sensitive to pre-clinical manifestations of AD [44]. This is important as neuro-cognitive changes occur many years prior to clinical manifestation of impaired physical function in AD and dementia [43]. Notably, we cannot preclude the potential influence of heritable or lifestyle factors which may have influenced these outcomes, for example an association between lower premorbid intelligence and early mortality [45]. Age stratification of females did not show any differences in the association between STW and slower physical function according to BMI classifications. Our findings build on prior evidence by highlighting the importance of maintaining physical function for cognitive health in females across mid and late adulthood, regardless of BMI classification. However, STW is a test of verbal intelligence shown to better represent predictive diagnostic ability in females which may partially explain why these findings were not observed in males in our study.

A novel outcome of our study was observed for males in late-adulthood (> 65 years), whereby obesity was associated with higher MMSE (reflective of lower dementia risk) and CVLT (reflective of better verbal memory) with slower physical function (reflective of poorer performance), compared to normal and overweight categories. These outcomes are consistent with the view that obesity in older ages is associated with reduced risk of dementia, yet this was only observed in males. Sex differences in body fat distribution and body composition may contribute to the sex-specific disparity in outcomes for physical function and MMSE in obese participants. The greater contribution of muscle and fat mass to BMI for males and females respectively [46], may add to greater disability in obese females and therefore poorer comparative physical function. Additionally, age-differences showing lower scores with increasing age as measured using the MMSE were greater in females than in males after age 75 years in a large cohort study (*n* = 13,004) [47]. The impact of obesity to sex and age-specific hormonal changes may also differ across cognitive domains [48]. For example, in males aged ≥ 60 years, greater verbal memory has been correlated with higher endogenous levels of oestradiol [49]. Although our results showed declines in the test for verbal memory for all BMI classifications as physical function slowed, the effect appeared attenuated only in obese males aged > 65 years. This may reflect obesity-induced increases in endogenous estrogen [48], however sex hormones were not measured in this study. Importantly, beneficial effects of maintaining mobility may attenuate potentially adverse obesity related effects to cognitive health in older males, however further research is needed to confirm or refute these findings.

To our knowledge, our study is the first to identify specific differences in the degree to which sex, age and BMI classification interact with physical function and specific measures of cognition. Measures of physical performance incorporating aspects of gait speed, dynamic balance/agility and lower limb strength may pose as multidimensional indicators of overall functional capacity and health status in older adults, yet decrease with ageing, obesity and diseases known to affect cognition [50, 51]. Measures of physical function (using the short physical performance battery) were positively associated with age-specific MMSE scores in a cohort study of healthy adults aged > 65 years adults (n = 102; mean (SD) age: 74 years, mean (SD) BMI: 28.3 [4.0] kg/m^2^) Post-hoc exploratory analyses of our outcomes showed that those who demonstrated impaired physical function (as determined by comparison to age-based norms i.e., 5% of the total sample, *n* = 118) were more likely to be overweight or obese according to either BMI classifications or waist circumference. Furthermore, in participants with normal cognition (MMSE ≥ 24 points) the median time to complete the physical function test differed by BMI classification (normal: 5.66 s, overweight: 5.72 s, obese: 6.26 s). These outcomes suggest that BMI classification may be a useful additional correlate to a model screening for dementia status (MMSE) in older males, however, further investigation is needed.

One prior study has examined the predictive ability of BMI classification, strength, physical function and cognition in older adults (*n* = 331, adults aged ≥ 55 years, mean age 77, 95% female), which included age stratification (young-old: aged 55–74 years versus old: aged > 75 years) [52]. This study noted no associations or interactions of BMI classification with grip strength or physical function (measured using the physical performance battery) [52]. However better cognitive flexibility (measured by Trail Making Test-B) was observed in obese adults aged ≥ 55 years after adjustment for self-perceived health, depressive symptoms, and number of medications [52]. In our study, analyses focussed on the degree to which BMI classification affected the interactions between strength, physical function, and cognition, (rather than independent effects). This may in part explain an apparent disparity in our findings with regards to associations and interactions of physical function and cognition noted in prior studies [52]. Furthermore, comparisons are limited due to differences in our measures used for cognition, strength and physical function, sample size and composition (56% of our sample were female) and adjustment for additional covariates (sex, cardiometabolic and comorbid risk factors, education, alcohol intake and physical activity) (48).

### Strengths and limitations

Our study was strengthened by including a large, population-based sample of Australian males and females aged 50 years and over. Moreover, direct and self-report measures of cardiometabolic disease, and other potential confounders, such as level of education, smoking history, perceived HR-QoL and physical activity (self-reported) were considered. The limitations of our study, however, should be considered. Prior studies have indicated that the relative risk of mortality and morbidity in obese adults is lower for individuals aged ≥ 65 years compared to younger adults, yet a stable or changing weight trajectory across adulthood may be more meaningful predictor of dementia risk [53]. Additionally, the binary nature of the age-stratification analyses may not sufficiently account for a confounding effect of a linear association between age and cognitive ability in contrast to age-adjusted associations. The cross-sectional design of this study precludes any inference of causality in the relationships between obesity, physical function and cognitive function in older adults and there was significant attrition from the sample prior to this wave of data collection, which would have introduced bias.

Future studies examining an interaction of BMI classification with physical and cognitive function might consider mechanistic effects of vascular health, weight trajectory and hormonal influences across older adulthood longitudinally. Participants included in the final study sample had a lower prevalence of depressive symptoms, T2D, smoking rates and showed higher education and PA levels than those of the overall cohort who participated in the larger 2011/2012 AusDiab wave, indicating a potential selection bias in those who completed strength and functional testing. Whilst these observations suggest that certain population groups, such as those with comparably lower socioeconomic status or greater prevalence of severe illness, may have been excluded from our analyses, this remains speculative.

## Conclusion

Our study demonstrates common patterns of reduction in cognitive and physical function with increasing chronological age. Novel findings were that obese females aged ≥ 50 years showed significantly better verbal intelligence (STW), and obese males aged > 65 years better verbal memory (CVLT) and general cognition (MMSE) with slower physical function compared to normal and overweight participants. Associations between lower limb muscle strength, and cognitive function in this study of Australian males and females were not influenced by BMI, sex or age category. These results highlight the importance of optimal physical function for cognitive health in older adults. Our findings also suggest BMI-based obesity indices may not consistently reflect physical and cognitive health in adults > 50 years and specifically in late adulthood (> 65 years) for males. Further investigations are needed including longitudinal follow-up of associations to establish whether these trends occur and continue as cohorts’ age.

## Supplementary Information

**Additional file 1. Supplementary Table 1**. Summary statistics for each of the cognition test in males and female participants in participants classified as normal weight (BMI <25kg/ m^2^), overweight (BMI ≥25 and <30kg/m^2^) and obese (BMI ≥ 30kg/m^2^). **Supplementary Table 2**. Multiplicative factor (95% CI) by which the odds of scoring an additional point in the cognitive test change given a 1kg increase in muscle strength (1 kg knee extensor test) for normal, overweight and obese participants according to sex and age categories. **Supplementary Table 3**. Multiplicative factor (95% CI) by which the odds of scoring an additional point in the cognitive test change given a 1-second increase in the physical function test (TUG) for normal, overweight and obese participants according to sex and age categories.

## Abbreviations

AD: Alzheimer's disease
AusDiab: Australian Diabetes, Obesity and Lifestyle study
BMI: Body mass index
CESD: Centre for Epidemiological Studies Depression Scale
CVD: Cardiovascular disease
CVLT: California verbal learning test
FFQ: Food frequency questionnaire
HR-QoL: Health-related quality of life
KES: Knee extensor strength
MCS: Mental component summary score
MMSE: Mini-mental-state exam
OR: Odds ratio
PA: Physical activity
PCS: Physical component summary score
PG: Plasma glucose
SDMT: Symbol-digit-modalities test
STW: Spot-the-word
T2D: Type 2 diabetes
TAFE: Technical and further education
TUG: Timed up-and-go
