# Supplementary material for: The influence of adiposity on the interactions between strength, physical function and cognition among older adults in the Australian Diabetes, Obesity and Lifestyle (AusDiab) study
Source: BMC Geriatr. 2022 Apr 22;22:357. doi: 10.1186/s12877-022-03033-3 (PMC9034532; doi:10.1186/s12877-022-03033-3)
Supplement: Supplementary file 1 — Additional file 1. Supplementary Table 1. Summary statistics for each of the cognition test in males and female participants in participants classified as normal weight (BMI <25kg/ m2), overweight (BMI ≥25 and <30kg/m2) and obese (BMI ≥ 30kg/m2). Supplementary Table 2. Multiplicative factor (95% CI) by which the odds of scoring an additional point in the cognitive test change given a 1kg increase in muscle strength (1 kg knee extensor test) for normal, overweight and obese participants according to sex and age categories. Supplementary Table 3. Multiplicative factor (95% CI) by which the odds of scoring an additional point in the cognitive test change given a 1-second increase in the physical function test (TUG) for normal, overweight and obese participants according to sex and age categories. [file 12877_2022_3033_MOESM1_ESM.docx]

**Supplementary Table 1.** Summary statistics for each of the cognition tests in males and female participants classified as normal weight (BMI <25kg/ m^2^), overweight (BMI ≥25 and <30kg/m^2^) and obese (BMI ≥ 30kg/m^2^).

|  | **BMI Category** | **Verbal Memory (CVLT)** | **Cognitive Reserve (STW)** | **Processing Speed (SDMT)** | **General Cognition (MMSE)** |
| --- | --- | --- | --- | --- | --- |
| **Male** | Normal weight | 5.7 ± 2.2  (95% CI, 5.4, 6.0)  N=260 | 50.7 ± 5.3  (95% CI, 50.0, 51.3)  N=257 | 47.9 ± 10.0  (95% CI, 46.7, 49.2)  N=261 | 28.0 ± 1.7  (95% CI, 27.7, 28.4)  N=115 |
|  | Overweight | 5.8 ± 2.2  (95% CI, 5.6, 6.0)  N=505 | 49.8 ± 6.0  (95% CI, 49.3, 50.3)  N=492 | 47.5 ± 9.5  (95% CI, 46.7, 48.4)  N=502 | 27.8 ± 2.1  (95% CI, 27.5, 28.1)  N=216 |
|  | Obese | 5.8 ± 2.1  (95% CI, 5.6, 6.1)  N=272 | 49.1 ± 6.1  (95% CI, 48.4, 49.9)  N=268 | 47.5 ± 10.5  (95% CI, 46.2, 48.7)  N=272 | 27.9 ± 2.0  (95% CI, 27.6, 28.3)  N=111 |
|  | P-value | 0.761 | **0.011** | 0.829 | 0.558 |
| **Female** | Normal weight | 7.3 ± 2.4  (95% CI, 7.0, 7.5)  N=419 | 50.7 ± 5.3  (95% CI, 50.2, 51.2)  N=413 | 51.2 ± 10.0  (95% CI, 50.3, 52.2)  N= 418 | 28.6 ± 1.5  (95% CI, 28.4, 28.9)  N=166 |
|  | Overweight | 6.8 ± 2.3  (95% CI, 6.6, 7.0)  N=502 | 50.2 ± 5.7  (95% CI, 49.7, 50.7)  N=498 | 50.6 ± 10.4  (95% CI, 49.7, 51.5)  N= 507 | 28.7 ± 1.5  (95% CI, 28.5, 28.9)  N=195 |
|  | Obese | 6.9 ± 2.3  (95% CI, 6.7, 7.2)  N=379 | 50.1 ± 6.1  (95% CI, 49.5, 50.7)  N=371 | 49.7 ± 10.8  (95% CI, 48.6, 50.8)  N= 381 | 28.1 ± 2.4  (95% CI, 27.8, 28.5)  N=164 |
|  | P-value | **0.012** | 0.319 | 0.117 | **0.009** |

Data are mean ± SD and 95% CI; N= number; CVLT: California Verbal Learning Test (n=2,337), MMSE: Mini Mental State Exam (n=967), SDMT: Symbol–Digit Modalities Test (n=2,341), STW: Spot-the-Word (n=2,299). A significant (p-value<0.05) indicates that there is a difference in the cognition tests amongst the BMI categories. Total sample: N=2337; Male: N=1037; Female: N= 1300

**Supplementary Table 2.** Multiplicative factor (95% CI) by which the predicted odds of scoring in the cognitive test change given a 1kg increase in muscle strength (1 kg knee extensor test) for normal, overweight and obese participants according to sex and age categories.

|  | Adjusted odds ratio at the means for the BMI categories | | | | | |
| --- | --- | --- | --- | --- | --- | --- |
|  | **Male** | **Female** | **Male, 50-65yr** | **Male, >65yr** | **Female, 50-65yr** | **Female, >65yr** |
| **Verbal Memory (California Verbal Learning Test)** | | | | | | |
| Normal | 1.000  (0.995, 1.006) | 1.002  (0.996, 1.008) | 1.003  (0.997, 1.010) | 1.001  (0.990, 1.013) | 1.001  (0.993, 1.008) | 1.009  (0.997, 1.020) |
| Overweight | 1.000  (0.996, 1.004) | 1.001  (0.996, 1.006) | 1.002  (0.998, 1.006) | 1.000  (0.992, 1.008) | 0.999  (0.993, 1.005) | 1.003  (0.994, 1.012) |
| Obese | 1.003  (0.997, 1.008) | 1.002  (0.996, 1.008) | 1.000  (0.994, 1.006) | **1.009**  **(1.000, 1.018)** | 1.004  (0.997, 1.012) | 0.998  (0.986, 1.009) |
| **Cognitive Reserve (Spot-the-Word)** | | | | | | |
| Normal | 1.000  (0.993, 1.006) | 1.002  (0.995, 1.009) | 0.999  (0.992, 1.006) | 1.000  (0.987, 1.013) | 1.002  (0.994, 1.010) | 0.999  (0.985, 1.013) |
| Overweight | 1.000  (0.995, 1.003) | 1.000  (0.995, 1.006) | 0.998  (0.994, 1.003) | 0.998  (0.989, 1.006) | 0.997  (0.991, 1.003) | 1.005  (0.994, 1.017) |
| Obese | 1.003  (0.997, 1.008) | 1.001  (0.994, 1.008) | 1.003  (0.996, 1.009) | 1.003  (0.993, 1.013) | 1.002  (0.994, 1.010) | 1.000  (0.985, 1.014) |
| **Processing Speed (Symbol–Digit Modalities Test)** | | | | | | |
| Normal | 1.000  (0.997, 1.003) | 1.000  (0.997, 1.004) | 1.002  (0.999, 1.006) | 0.997  (0.992, 1.003) | 1.002  (0.998, 1.007) | 1.002  (0.994, 1.010) |
| Overweight | 1.001  (0.999, 1.003) | 1.002  (0.999, 1.005) | 1.002  (0.999, 1.004) | 1.003  (0.999, 1.007) | 1.002  (0.999, 1.006) | 1.002  (0.996, 1.008) |
| Obese | 1.001  (0.998, 1.003) | 1.002  (0.999, 1.005) | 1.000  (0.999, 1.004) | 1.003  (0.998, 1.007) | 1.004  (1.000, 1.008) | 1.002  (0.995, 1.010) |
| **General Cognition (Mini Mental State Exam)** | | | | | | |
| Normal | 0.994  (0.979, 1.009) | 0.985  (0.966, 1.004) | 0.993  (0.964, 1.022) | 0.995  (0.976, 1.014) | 0.976  (0.949, 1.003) | 0.995  (0.971, 1.020) |
| Overweight | 1.000  (0.989, 1.011) | 0.997  (0.979, 1.015) | 0.999  (0.983, 1.015) | 1.003  (0.989, 1.017) | 0.987  (0.958, 1.016) | 1.002  (0.979, 1.025) |
| Obese | 0.987  (0.974, 1.000) | **0.981**  **(0.963, 0.999)** | 0.978  (0.956, 1.001) | 0.992  (0.976, 1.008) | 0.985  (0.958, 1.011) | 0.983  (0.959, 1.007) |

The results presented are OR (95%CI) for Model C adjusted for sex, age, smoking status, total physical activity, cardiovascular disease risk, type 2 diabetes status, education, depression, alcohol intake, SF-36 mental and physical component scores. **P<0.050**. The sample size for each stratification is given below.

**Verbal memory test -**Total sample: 2337; Male: 1037; Female: 1300; Male 50-65 yrs: 615; Male >65 yrs: 422; Female 50-65 yrs: 800; Female >65 yrs: 500

**Spot-the-Word -**Total sample: 2299; Male: 1017; Female: 1282; Male 50-65 yrs: 607; Male >65 yrs: 410; Female 50-65 yrs: 792; Female >65 yrs: 490

**Symbol–Digit Modalities Test -**Total sample: 2341; Male: 1035; Female: 1306; Male 50-65 yrs: 614; Male >65 yrs: 421; Female 50-65 yrs: 802; Female >65 yrs: 504

**Mini Mental State Exam -**Total sample: 967; Male: 442; Female: 525; Male 50-65 yrs: 154; Male >65 yrs: 288; Female 50-65 yrs: 171; Female >65 yrs: 354

**Supplementary Table 3.** Multiplicative factor (95% CI) by which the predicted odds of scoring in the cognitive test change given a 1-second increase in the physical function test (TUG) for normal, overweight and obese participants according to sex and age categories.

|  | Adjusted odds ratio at the means for the BMI categories | | | | | |
| --- | --- | --- | --- | --- | --- | --- |
|  | **Male** | **Female** | **Male, 50-65yr** | **Male, >65yr** | **Female, 50-65yr** | **Female, >65yr** |
| **Verbal Memory (CVLT)** | | | | | | |
| Normal | **0.922**  **(0.882, 0.961)** | **0.957**  **(0.915, 0.998)** | 0.965  (0.906, 1.025) | **0.874**  **(0.814, 0.933)** | **0.926**  **(0.865, 0.986)** | 0.977  (0.917, 1.036) |
| Overweight | 0.969  (0.930, 1.008) | 0.986  (0.949, 1.024) | 0.967  (0.915, 1.019) | **0.929**  **(0.871, 0.987**) | 0.985  (0.926, 1.044) | 0.988  (0.935, 1.041) |
| Obese | 0.973  (0.937, 1.010) | 0.985  (0.957, 1.012) | 0.970  (0.908, 1.031) | 0.981  (0.932, 1.029) | **0.946**  **(0.894, 0.998)** | 0.999  (0.966, 1.032) |
| **Cognitive Reserve (Spot-the-Word)** | | | | | | |
| Normal | 0.965  (0.926, 1.004) | **0.922**  **(0.880, 0.964)** | 0.981  (0.917, 1.046) | 0.956  (0.902, 1.01) | **0.932**  **(0.867, 0.997)** | **0.927**  **(0.863, 0.990)** |
| Overweight | 0.996  (0.952, 1.041) | 0.971  (0.930, 1.013) | 0.998  (0.939, 1.057) | 1.016  (0.943, 1.088) | 0.996  (0.936, 1.057) | 0.975  (0.912, 1.040) |
| Obese | 0.991  (0.955, 1.026) | 0.987  (0.958, 1.015) | 1.001  (0.934, 1.068) | 0.986  (0.940, 1.031) | 1.015  (0.957, 1.073) | 0.984  (0.949, 1.020) |
| **Processing Speed (Symbol–Digit Modalities Test)** | | | | | | |
| Normal | **0.958**  **(0.937, 0.978)** | **0.973**  **(0.949, 0.998)** | **0.945**  **(0.911, 0.978)** | **0.969**  **(0.941, 0.998)** | 0.969  (0.933, 1.004) | 0.962  (0.922, 1.001) |
| Overweight | **0.958**  **(0.938, 0.979)** | **0.962**  **(0.941, 0.984)** | **0.943**  **(0.914, 0.972)** | **0.936**  **(0.907, 0.966)** | **0.954**  **(0.922, 0.986)** | **0.961**  **(0.927, 0.995)** |
| Obese | **0.968**  **(0.949, 0.987)** | **0.971**  **(0.955, 0.987)** | 0.969  (0.934, 1.004) | **0.973**  **(0.949, 0.997)** | **0.949**  **(0.920, 0.978)** | **0.967**  **(0.945, 0.989)** |
| **General Cognition (Mini Mental State Exam)** | | | | | | |
| Normal | **0.880**  **(0.802, 0.957)** | 0.939  (0.841, 1.037) | 1.029  (0.791, 1.268) | **0.846**  **(0.764, 0.927)** | 0.890  (0.668, 1.112) | 0.951  (0.836, 1.066) |
| Overweight | **0.869**  **(0.791, 1 0.946)** | 0.947  (0.847, 1.047) | 0.865  (0.695, 1.035) | **0.839**  **(0.756, 0.923)** | 0.912  (0.708, 1.115) | 0.938  (0.820, 1.055) |
| Obese | 1.018  (0.908, 1.127) | 0.947  (0.883, 1.011) | 0.988  (0.824, 1.152) | 1.043  (0.902, 1.185) | 0.971  (0.801, 1.140) | 0.939  (0.867, 1.012) |

The results presented are OR (95%CI) for Model C adjusted for sex, age, smoking status, total physical activity, cardiovascular disease risk, type 2 diabetes status, education, depression, alcohol intake, SF-36 mental and physical component scores. **P<0.050**. The sample size for each stratification is given below.

**Verbal memory test -**Total sample: 2337; Male: 1037; Female: 1300; Male 50-65 yrs: 615; Male >65 yrs: 422; Female 50-65 yrs: 800; Female >65 yrs: 500

**Spot-the-Word -**Total sample: 2299; Male: 1017; Female: 1282; Male 50-65 yrs: 607; Male >65 yrs: 410; Female 50-65 yrs: 792; Female >65 yrs: 490

**Symbol–Digit Modalities Test -**Total sample: 2341; Male: 1035; Female: 1306; Male 50-65 yrs: 614; Male >65 yrs: 421; Female 50-65 yrs: 802; Female >65 yrs: 504

**Mini Mental State Exam -**Total sample: 967; Male: 442; Female: 525; Male 50-65 yrs: 154; Male >65 yrs: 288; Female 50-65 yrs: 171; Female >65 yrs: 354
